# Plurality of Primary and Specialty Care in Older Adults With Dementia and Multimorbidity

**DOI:** 10.1093/geroni/igaf122.3642

**Published:** 2025-12-31

**Authors:** Sijia Wei, Eleanor McConnell, Daniela Ladner, Blade Robelly, Jeffrey Linder

**Affiliations:** Northwestern University, Chicago, Illinois, United States; Duke University, Durham, North Carolina, United States; Northwestern University Feinberg School of Medicine, Chicago, Illinois, United States; Northwestern University Feinberg School of Medicine, Chicago, Illinois, United States; Northwestern University Feinberg School of Medicine, Chicago, Illinois, United States

## Abstract

Older adults with comorbidities often require care across specialties. Those with Alzheimer’s Disease and Related Dementias (ADRD) face additional challenges that require coordinated outpatient care. However, the plurality of primary care (PC) and specialty use among this population remains understudied. This retrospective cohort study used electronic health records from a multi-site academic health system in the Chicago metropolitan area. The cohort included older adults (≥65 years) with ≥2 chronic conditions and regular outpatient visits between 2016-2024, stratified by ADRD diagnosis. Outpatient encounters were classified into PC (internal/family medicine), ADRD specialty (geriatrics, neurology, psychiatry), or other specialty. Patients were categorized based on whether their care was PC-dominant (> mean proportion of PC encounters) and whether they accessed any ADRD specialty. Among 64,279 patients, 16.2% had ADRD. Of 10,687,974 outpatient encounters, only 11.2% were PC. Overall, 18.6% of patients never accessed PC, more often among those without ADRD (21.6%) than those with (16.8%). Among patients with any PC, the average proportion of PC encounters was 16.7%±13.3%. Patients with ADRD had less specialty-dominant care (50.0% vs. 61.1%). Regardless of ADRD status (with ADRD vs without), patients without PC had the lowest ADRD-specialty use (55% vs 26%), while those with specialty-dominant care had the highest (78% vs 42%). Survival was highest among patients with PC-dominant care (57% with ADRD; 81% without ADRD). These findings highlight distinct outpatient care patterns and potential coordination gaps among PC and specialty care that need further study, which may impact outcomes among older adults with ADRD and multimorbidity.

